# Endotoxin Induces Fibrosis in Vascular Endothelial Cells through a Mechanism Dependent on Transient Receptor Protein Melastatin 7 Activity

**DOI:** 10.1371/journal.pone.0094146

**Published:** 2014-04-07

**Authors:** Cesar Echeverría, Ignacio Montorfano, Tamara Hermosilla, Ricardo Armisén, Luis A. Velásquez, Claudio Cabello-Verrugio, Diego Varela, Felipe Simon

**Affiliations:** 1 Departamento de Ciencias Biologicas, Facultad de Ciencias Biologicas and Facultad de Medicina, Universidad Andres Bello, Santiago, Chile; 2 Millennium Institute on Immunology and Immunotherapy, Santiago, Chile; 3 Centro de Estudios Moleculares de la Celula, Facultad de Medicina, Universidad de Chile, Santiago, Chile; 4 Instituto de Ciencias Biomedicas, Facultad de Medicina, Universidad de Chile, Santiago, Chile; 5 Centro de Investigacion y Tratamiento del Cancer, Facultad de Medicina, Universidad de Chile, Santiago, Chile; 6 Center for Integrative Medicine and Innovative Science (CIMIS), Facultad de Medicina, Universidad Andres Bello, Santiago, Chile; 7 Centro para el Desarrollo de la Nanociencia y Nanotecnología, Universidad de Santiago de Chile, Santiago, Chile; Virginia Polytechnic Institute and State University, United States of America

## Abstract

The pathogenesis of systemic inflammatory diseases, including endotoxemia-derived sepsis syndrome, is characterized by endothelial dysfunction. It has been demonstrated that the endotoxin lipopolysaccharide (LPS) induces the conversion of endothelial cells (ECs) into activated fibroblasts through endothelial­to­mesenchymal transition mechanism. Fibrogenesis is highly dependent on intracellular Ca^2+^ concentration increases through the participation of calcium channels. However, the specific molecular identity of the calcium channel that mediates the Ca^2+^ influx during endotoxin-induced endothelial fibrosis is still unknown. Transient receptor potential melastatin 7 (TRPM7) is a calcium channel that is expressed in many cell types, including ECs. TRPM7 is involved in a number of crucial processes such as the conversion of fibroblasts into activated fibroblasts, or myofibroblasts, being responsible for the development of several characteristics of them. However, the role of the TRPM7 ion channel in endotoxin-induced endothelial fibrosis is unknown. Thus, our aim was to study whether the TRPM7 calcium channel participates in endotoxin-induced endothelial fibrosis. Using primary cultures of ECs, we demonstrated that TRPM7 is a crucial protein involved in endotoxin-induced endothelial fibrosis. Suppression of TRPM7 expression protected ECs from the fibrogenic process stimulated by endotoxin. Downregulation of TRPM7 prevented the endotoxin-induced endothelial markers decrease and fibrotic genes increase in ECs. In addition, TRPM7 downregulation abolished the endotoxin-induced increase in ECM proteins in ECs. Furthermore, we showed that intracellular Ca^2+^ levels were greatly increased upon LPS challenge in a mechanism dependent on TRPM7 expression. These results demonstrate that TRPM7 is a key protein involved in the mechanism underlying endotoxin-induced endothelial fibrosis.

## Introduction

Sepsis syndrome is the most prevalent cause of mortality in critically ill patients admitted to intensive care units [1]. The pathogenesis of sepsis syndrome develops through an overactivation of the immune system, which involves activation of immune cells, secretion of pro-inflammatory cytokines and generation of reactive oxygen species (ROS) [1], [2]. Despite numerous basic and clinical studies addressing sepsis syndrome, current therapies for treating it and its sequelae are unsatisfactory, exhibiting high morbimortality rates [3], [4].

Endotoxemia-derived sepsis syndrome is a important cause of sepsis syndrome. It is frequently characterized by deposition of large amounts of the Gram-negative bacterial endotoxin, lipopolysaccharide (LPS) [5]–[8]. During endotoxemia, the endotoxin circulating in the bloodstream interacts with the endothelial cells (ECs) located in the internal endothelial monolayer of blood vessels, inducing detrimental effects on endothelium function [9]–[11].

It is well accepted that the endothelial dysfunction is an important factor in the pathogenesis of endotoxemia-derived sepsis syndrome as well as other inflammatory diseases [9], [12]. We reported that LPS induces at least two main effects in vascular ECs. First, endotoxin promotes endothelial cell death [13]. Second, LPS is able to induce the conversion of ECs into activated fibroblasts, also known as myofibroblasts [14]. Endotoxin-induced endothelial fibrosis is mediated through a process known as endothelial­to­mesenchymal transition (EndMT) in a similar way that observed using the best-studied EndMT inducers, tumor growth factor- beta 1 and 2 (TGF-β1 and TGF-β2) [15], [16]. Endotoxin-induced endothelial fibrosis is characterized by downregulation of the endothelial markers CD31/PECAM and VE-cadherin, whereas the fibroblast-specific genes α-smooth muscle actin (α-SMA) and fibroblast-specific protein 1 (FSP-1) are upregulated. Furthermore, the levels of proteins that form the extracellular matrix (ECM), such as fibronectin (FN) and type III collagen (Col III), are greatly increased [14].

It has been reported that Ca^2+^ influx is absolutely required for fibrosis development. The generation of myofibroblasts from cultured rat cardiac fibroblasts is inhibited by chelating external Ca^2+^ [17], [18], while decreasing the intracellular Ca^2+^ concentration improves liver and muscle fibrosis [19], [20]. Furthermore, increases in intracellular oxidative stress and pro-inflammatory cytokine synthesis and secretion, both of which are major features of fibrosis, are attenuated by inhibition of the Ca^2+^ influx [17], [18], [21]. In addition, increased cell migration, a distinctive attribute of activated fibroblasts, is also dependent on Ca^2+^ entry [22], [23]. Therefore, Ca^2+^ entry is an essential step in the development of the characteristics of fibrosis.

Determining the molecular entity that mediates the Ca^2+^ influx during fibrogenesis is an issue of great importance due to its therapeutic implications. It has been reported that L-type calcium channels modulate perivascular fibrosis in the kidney [24]. Similarly, it has been reported that blocking of T-type and L-type calcium channels is effective in decreasing tubulointerstitial fibrosis [25]. Furthermore, cardiac fibrosis was found to be decreased when calcium channel blockers were used in addition to complementary treatments [26], [27]. Accordingly, inhibition of calcium channels is effective in attenuating liver fibrogenesis [28]. These data suggest that calcium channels are required for Ca^2+^ influx to promote fibrosis. However, the molecular identity of the calcium channel that mediates the Ca^2+^ influx during endotoxin-induced endothelial fibrosis remains unknown.

It has been shown that transient receptor potential melastatin 7 (TRPM7) is the main Ca^2+^-permeable channel involved in the TGF-β1-induced activation of human atrial fibroblasts into myofibroblasts, promoting atrial fibrillation [29]. In addition, TRPM7 is involved in the migration of fibroblasts, which is a typical feature of myofibroblasts [30], [31]. The TRPM7 ion channel is permeable to the divalent cations Ca^2+^ and Mg^2+^ but is impermeable to monovalent cations. This channel is ubiquitously expressed in a broad range of cell types, including ECs [32]–[36]. These findings suggest that TRPM7 could be involved in the conversion of endothelial cells into myofibroblasts upon endotoxin challenge. However, the role of the TRPM7 ion channel in endotoxin-induced endothelial fibrosis is currently not known.

Therefore, the aim of this study was to investigate whether the TRPM7 protein is involved in endotoxin-induced endothelial fibrosis.

Our data demonstrated that the TRPM7 ion channel plays a crucial role in endotoxin-induced endothelial fibrosis. Suppression of TRPM7 expression protected ECs from the endotoxin-induced fibrogenic process. Additionally, we demonstrated that intracellular Ca^2+^ levels were increased in ECs exposed to endotoxin. This endotoxin-induced Ca^2+^ increase was abolished by inhibition of TRPM7 expression, suggesting that the TRPM7-mediated Ca^2+^ increase is involved in the mechanism underlying endotoxin-induced endothelial fibrosis.

The results provided here contribute to our understanding of the molecular basis of endotoxin-induced endothelial fibrosis, revealing a novel target that could be useful in drug development for treating endothelial dysfunction during endotoxemia and other inflammatory diseases.

## Materials and Methods

Details of all procedures are provided in Methods S1.

### Ethics Statement

The investigation conforms with the principles outlined in the Declaration of Helsinki. The Commission of Bioethics and Biosafety of Universidad Andres Bello also approved all experimental protocols. Human umbilical cord were obtained from patients after written patient's informed consent. The individual in this manuscript has given written informed consent (as outlined in PLOS consent form) to publish these case details.

### Primary cell culture

Human umbilical vein endothelial cells (HUVEC) were isolated by collagenase (0.25 mg/mL) digestion from freshly obtained umbilical cord veins from normal pregnancies, after patient's informed consent. Cells were grown in gelatin-coated dishes at 37°C in a 5%:95% CO_2_:air atmosphere in medium 199 (Sigma, MO), containing 100 μg/mL endothelial cell growth supplement (ECGS) (Sigma), 100 μg/mL heparin, 5 mmol/L D-glucose, 3.2 mmol/L L-gutamine, 10% fetal bovine serum (FBS) (GIBCO, NY), and 50 U/mL penicillin-streptomycin (Sigma).

### Small interfering RNA and transfection

SiGENOME SMARTpool siRNA (four separated siRNAs per human TRPM7 transcript) were purchased from Dharmacon (Dharmacon, Lafayette, CO). The following siRNA were used: human TRPM7 (siRNA-TRPM7) and non-targeting siRNA (siRNA-CTRL) used as a control. In brief, HUVEC were plated overnight in 24-well plate and then transfected with 5 nM siRNA using DharmaFECT 4 transfection reagent (Dharmacon) used according to the manufacturer's protocols in serum-free medium for 6 hours. After 48 to 72 h after transfection, experiments were performed.

### Western blot procedures

Vehicle-treated or endotoxin-treated ECs in non-transfected or transfected conditions were lysed in cold lysis buffer, and then proteins were extracted. Supernatants were collected and stored in the same lysis buffer. Protein extract and supernatant were subjected to SDS-PAGE and resolved proteins were transferred to a nitrocellulose or PVDF membrane. The blocked membrane was incubated with the appropriate primary antibody, washed twice, and incubated with a secondary antibody. Bands were revealed using a peroxidase-conjugated IgG antibody. Tubulin was used as a loading control. For a detailed list of antibodies used, see Table S1.

### Fluorescent Immunocytochemistry

ECs were washed twice with PBS and fixed. The cells were subsequently washed again and incubated with the primary antibodies. Then, cells were washed twice and incubated with the secondary antibodies. Samples were mounted with ProLong Gold antifade mounting medium with DAPI (Invitrogen). For a detailed list of antibodies used see Table S2.

### Calcium Imaging

Plated ECs were mounted in a perfusion chamber on the stage of an inverted microscope (Olympus IX-81, UPLFLN 40XO 40 x/1.3 oil-immersion objective). Cells were incubated with 1 μM Fura-2 AM (Molecular Probes) for 30 min and then washed with Hank's solution. ECs were transfected (with siRNA-TRPM7 and siRNA-CTRL) or non-transfected. Fura-2 was alternately excited at 340 and 400 nm, and the fluorescence filtered at 510 nm was collected and recorded at 5 Hz using a CCD-based imaging system (Olympus DSU) running CellR software (Olympus). At the end of each experiment, maximal fluorescence was obtained by treating the cell with 1 μM ionomicin. For every experiment, signals were recorded and the background intensity was subtracted, using a same-size region of interest outside the cells [37]. Results are expressed as the ratio between the 340 nm and 400 nm (R340/400) signals.

### Measurement of [Ca^2+^] by flow cytometry

ECs were harvested with trypsin/EDTA, washed twice in ice-cold PBS, resuspended and loaded with the Ca^2+^-sensitive cell permeant dye Fluo-4 (5 μM) for 15–30 min at room temperature in the dark. Then, cells were exposed to LPS for 90 s and analyzed immediately by flow cytometry (FACSCanto, BD Biosciences, San José, CA). ECs were transfected (with siRNA-TRPM7 and siRNA-CTRL) or preincubated with L-NAME, MCI-186, NAC, and GSH. Experiments were performed and then intracellular calcium levels were measured using Fluo-4 dye. Using the FACSDiva software, a population of red-positive cells (transfected cells) was defined, and calcium levels for this population were analyzed. A minimum of 10,000 cells/sample were analyzed. Cellular dye intensity analysis was performed using FACSDiva software v4.1.1 (BD Biosciences).

### Reagents

Lipopolysaccharide from *E. coli* was purchased from Sigma (0127:B8). Fura-2 and Fluo-4 were purchased from Invitrogen. L-NAME, cobinamide, NAC and GSH were purchased from Sigma. PTIO and L-NMMA were purchased from Tocris Bioscience (Bristol, UK). MCI-186 was purchased from Calbiochem (San Diego, CA). Human TGF-β1 and TGF-β2 were purchased from R&D Systems. Buffers and salts were purchased from Merck Biosciences (Darmstadt).

### Data analysis

All results are presented as the means ± SD. One-way analysis of variance (ANOVA) (Kruskal–Wallis) followed by Dunn's *post hoc* test were used and considered significant at p<0.05.

## Results

### Endothelial cells convert into activated fibroblasts upon endotoxin challenge

**Figure 1:**
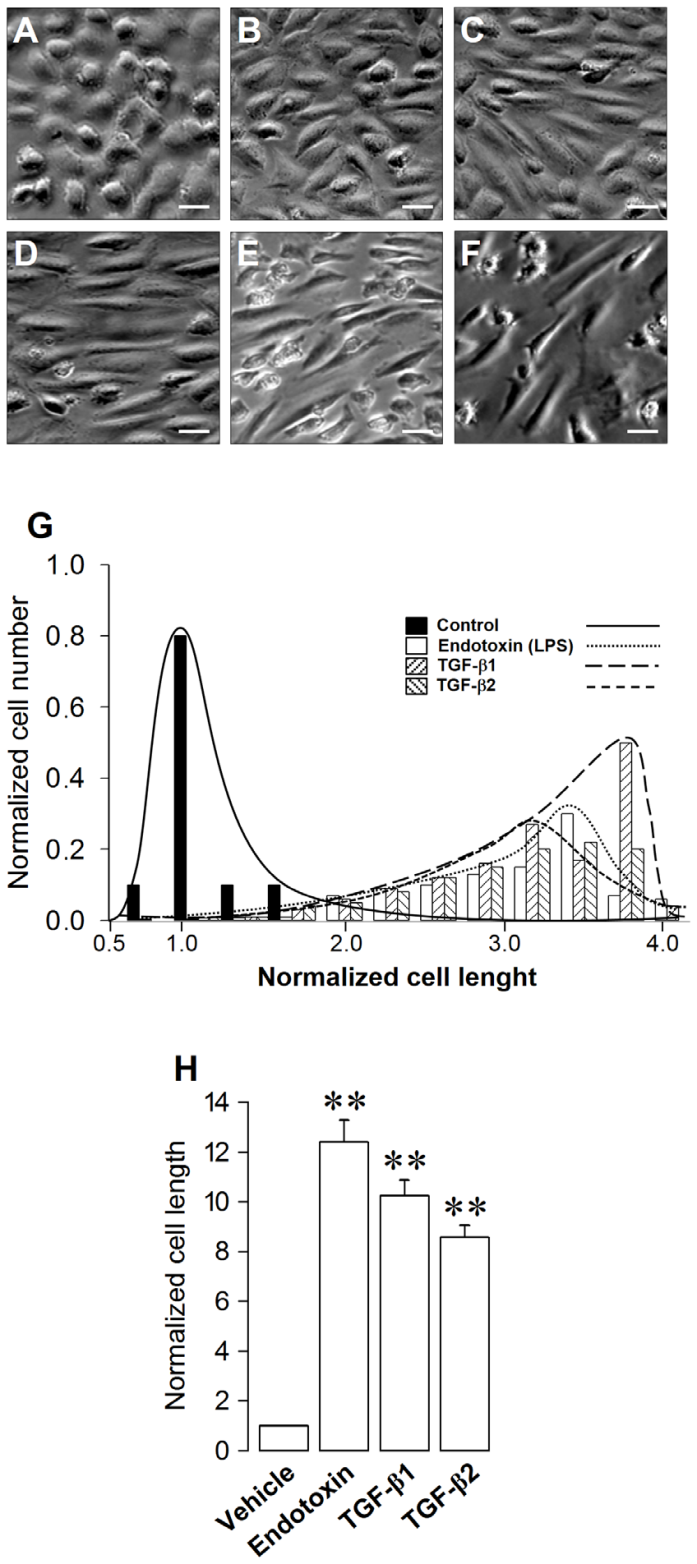
Endotoxin-induced fibroblast-like morphology in endothelial cells. (A–F) Morphological changes in human ECs resembling fibroblast. Figures show representative phase-contrast images from at least three separates experiments of non-transfected ECs exposed to vehicle (A), 20 μg/mL LPS for 24 h (B), 20 μg/mL LPS for 48 h (C), 20 μg/mL LPS for 72 h (D), 2.5 ng/mL TGF-β1 (E), 2.5 ng/mL TGF-β2, (F). Bar scale represents 50 μm. (G) Endothelial cell length distribution of cells exposed to vehicle (filled bars), 20 μg/mL LPS for 72 h (empty bars), 2.5 ng/mL TGF-β1 (dashed bars), and 2.5 ng/mL TGF-β2, (dashed bars). *N* = 3-6. (H) Endothelial cell length in which length/width >2, for cells exposed to vehicle, 20 μg/mL LPS for 72 h, 2.5 ng/mL TGF-β1, and 2.5 ng/mL TGF-β2. Statistical differences were assessed by a one-way analysis of variance (ANOVA) (Kruskal–Wallis) followed by Dunn's post hoc test. **: *p*<0.01 against the vehicle-treated condition. Graph bars show the mean ± SD (*N* = 3-6).

Endothelial cells normally exhibit a round, short-spindle morphology, with a cobblestone appearance (Figure 1A). In contrast, ECs exposed to the endotoxin LPS showed a spindle-shaped, fibroblast-like phenotype (Figure 1B–D), similar to what has been reported previously [14]. This phenotype is similar to that obtained using the best-studied EndMT inducers, TGF-β1 (Figure 1E) and TGF-β2 (Figure 1F), suggesting that LPS induces endothelial fibrosis through EndMT.

To study these phenotypic changes in detail, we measured the length of cells in the absence or presence of LPS. The results revealed a differential distribution of cell lengths in ECs in the presence of vehicle compared to cells exposed to LPS. The mean cell length of ECs exposed to LPS was ∼4-fold higher than observed in the absence of LPS. Interestingly, ECs exposed to the typical transforming inducers TGF-β1 and TGF-β2 exhibited a similar cell length distribution as LPS-treated cells (Figure 1G). Next, we counted only cells whose length was twice their width (length/width >2) because in the cell length analysis showed in Figure 1G, the length measurements were merged for a significant portion of the cells. The results showed that the length of endotoxin-treated cells was ∼12-fold higher than that of ECs in the absence of LPS. Similar results were obtained in ECs exposed to TGF-β1 and TGF-β2 (Figure 1H).

To demonstrate that our results were obtained from cultures of ECs without contamination from fibroblasts or mesenchymal-like cells, we performed a detailed examination of our EC cultures. Using VE-cadherin as a specific endothelial marker, we found that >99% of the cells in our EC cultures were positive for VE-cadherin, demonstrating that our primary EC cultures were highly enriched in ECs (Figure S1).

### TRPM7 expression is crucial for the alteration of endothelial and fibrotic marker expression induced by endotoxin in endothelial cells

**Figure 2:**
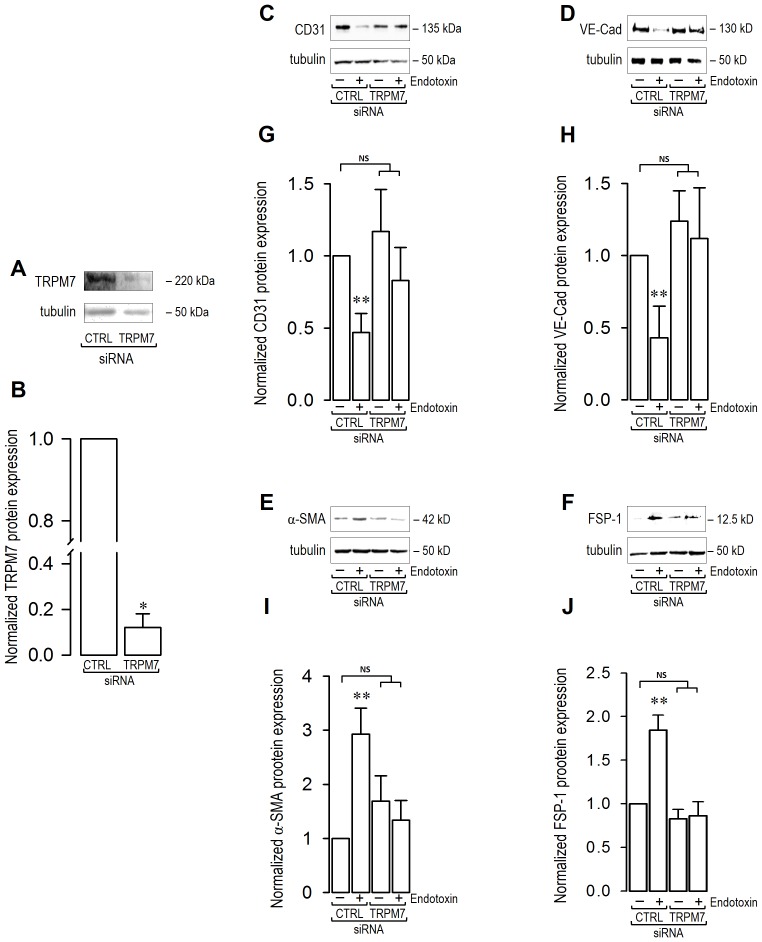
Endotoxin-induced endothelial fibrosis through changes in endothelial and fibrotic markers is dependent on TRPM7 expression. (A–B) TRPM7 expression downregulation by siRNA. ECs were transfected with a specific siRNA against the human TRPM7 isoform (siRNA-TRPM7) or a non-targeting siRNA (siRNA-CTRL). (A) Representative images from western blot experiments performed for detection of TRPM7 in cells transfected with siRNA-TRPM7 or siRNA-CTRL. (B) Densitometric analyses from several experiments, as shown in (A). Protein levels were normalized against tubulin, and the data are expressed relative to cells transfected with siRNA-CTRL condition. Statistical differences were assessed by student's t-test (Mann-Whitney). ***: *p*<0.001. Graph bars show the mean ± SD (*N* = 3). (C–J) ECs were exposed to LPS for 72 h and protein expression was analyzed. (C–F) Representative images from western blot experiments performed for detection of endothelial markers CD31 (C) and VE-cadherin (VE-Cad) (D), and fibrotic markers α-SMA (E) and FSP-1 (F). (G–J) Densitometric analyses of the experiments shown in (C–F) respectively. Protein levels were normalized against tubulin and data are expressed relative to siRNA-CTRL transfected cells without endotoxin condition. Statistical differences were assessed by a one-way analysis of variance (ANOVA) (Kruskal–Wallis) followed by Dunn's post hoc test. *: *p*<0.05 and **: *p*<0.01 against to siRNA-CTRL transfected cells without endotoxin condition. NS: non-significant. Graph bars show the mean ± SD (*N* = 3–6).

To test whether the TRPM7 channel is involved in endotoxin-induced endothelial fibrosis, we applied a molecular biological experimental strategy to achieve downregulation of TRPM7 expression. Thus, we used a specific small interfering RNA (siRNA) targeting the human isoform of TRPM7 (siRNA-TRPM7). The efficiency of the siRNA in achieving downregulation of TRPM7 channel expression was >90% compared to ECs transfected with a non-targeting siRNA sequence used as a control (siRNA-CTRL) (Figure 2A-B).

As expected, LPS-treated ECs transfected with siRNA-CTRL showed similar characteristics to those previously observed in non-transfected wild-type endothelial cells exposed to LPS [14]. ECs transfected with siRNA-CTRL and exposed to endotoxin exhibited a decrease in the expression of the endothelial proteins CD31 (Figure 2C and G) and VE-cadherin (Figure 2E and H). Furthermore, the protein expression of the fibrotic markers α-SMA (Figure 2E and I) and FSP-1 (Figure 2F and J) was significantly increased upon endotoxin challenge. In contrast, LPS-treated ECs transfected with siRNA-TRPM7 were resistant to endotoxin challenge. ECs transfected with siRNA-TRPM7 did not show any decrease in the expression of the endothelial proteins CD31 (Figure 2C and G) and VE-cadherin (Figure 2E and H). Accordingly, the levels of the fibrotic markers α-SMA (Figure 2E and I) and FSP-1 (Figure 2F and J) were not increased in LPS-treated ECs transfected with siRNA-TRPM7. ECs transfected with siRNA-TRPM7 in the absence of LPS did not show any difference in the expression of endothelial and fibrotic markers compared to that observed in vehicle-treated cells transfected with siRNA-CTRL (Figure 2C–J). Similar results were founded using the non-specific TRPM7 blockers Zn^2+^ and Gd^3+^ (Figure S2) [33], [38]–[40].

**Figure 3:**
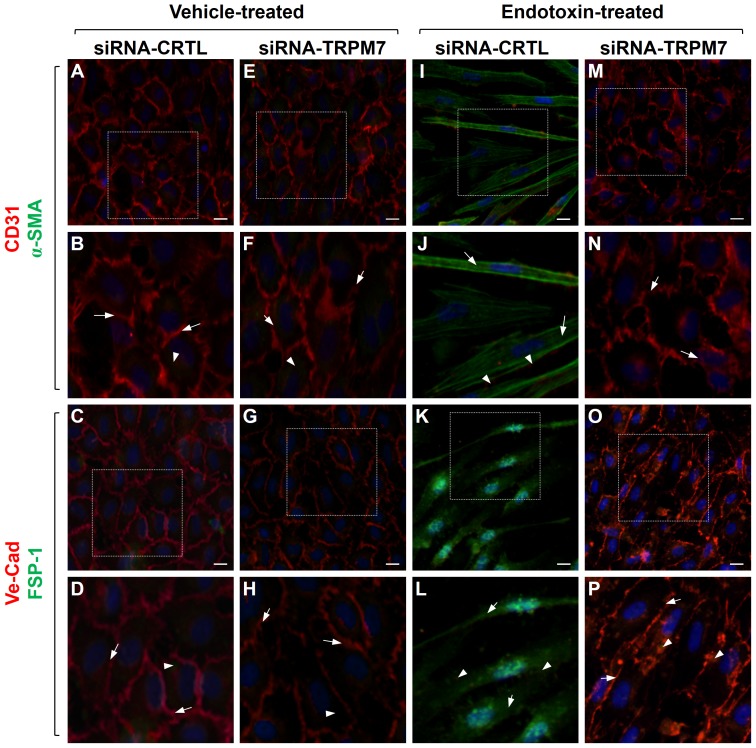
Cellular distribution of endothelial and fibrotic markers involved in endotoxin-induced endothelial fibrosis. Representative images from experiments of vehicle-treated (A–H) or endotoxin (20 μg/mL LPS)-treated (I–P) ECs for 72 h. Endothelial markers CD31 or VE-cadherin (red), and the fibrotic markers α-SMA, or FSP-1 (green) were detected. In vehicle-treated cells: the box depicted in (A, C, E, and G) indicates the magnification shown in (B, D, F, and H), respectively. Arrows indicate CD31 (B, F) or VE-cadherin (D, H) labeling at the plasma membrane, whereas arrowheads indicate α-SMA (B, F) or FSP-1 (D, H) staining, indicating basal expression of fibrotic markers (B, D). In endotoxin-treated cells: the box depicted in (I, K, M, and O), indicates the magnification shown in (J, L, N, and P) respectively. Arrows indicate α-SMA (J, N) or FSP-1 (L. P), whereas arrowheads indicate CD31 (J, N) or VE-cadherin (L, P) staining from residual endothelial marker expression indicating EndMT. Nuclei were stained using DAPI. Bar scale represents 10 μm.

To study the participation of TRPM7 in the cellular localization and distribution of endothelial and fibrotic proteins, we carried out immunocytochemistry experiments. ECs transfected with siRNA-CTRL or siRNA-TRPM7 in the absence of endotoxin showed typical CD31 labeling, localized predominantly to the plasma membrane, whereas α-SMA was weakly expressed (Figure 3A and E). VE-cadherin labeling was also detected at the plasma membrane, while FSP-1 expression was undetectable (Figure 3C and G). ECs transfected with siRNA-CTRL and exposed to LPS showed increased α-SMA labeling in fibrotic-like stress fibers and decreased CD31 expression (Figure 3I). Additionally, FSP-1 labeling was greatly increased, whereas VE-cadherin was virtually absent (Figure 3K). This distribution was analogous to that previously reported in LPS-treated, non-transfected wild-type endothelial cells [14]. Conversely, endotoxin-treated ECs transfected with siRNA-TRPM7 did not show changes in the localization and distribution of endothelial and fibrotic markers, showing similar results to those observed in the absence of LPS. Endotoxin-treated ECs transfected with siRNA-TRPM7 exhibited CD31 localized mainly at the plasma membrane, while α-SMA was weakly detected (Figure 3M). Moreover, VE-cadherin was also detected at the plasma membrane, but FSP-1 expression was undetectable (Figure 3O).

### TRPM7 expression is crucial for the increase of extracellular matrix proteins induced by lipopolysaccharide in endothelial cells

**Figure 4:**
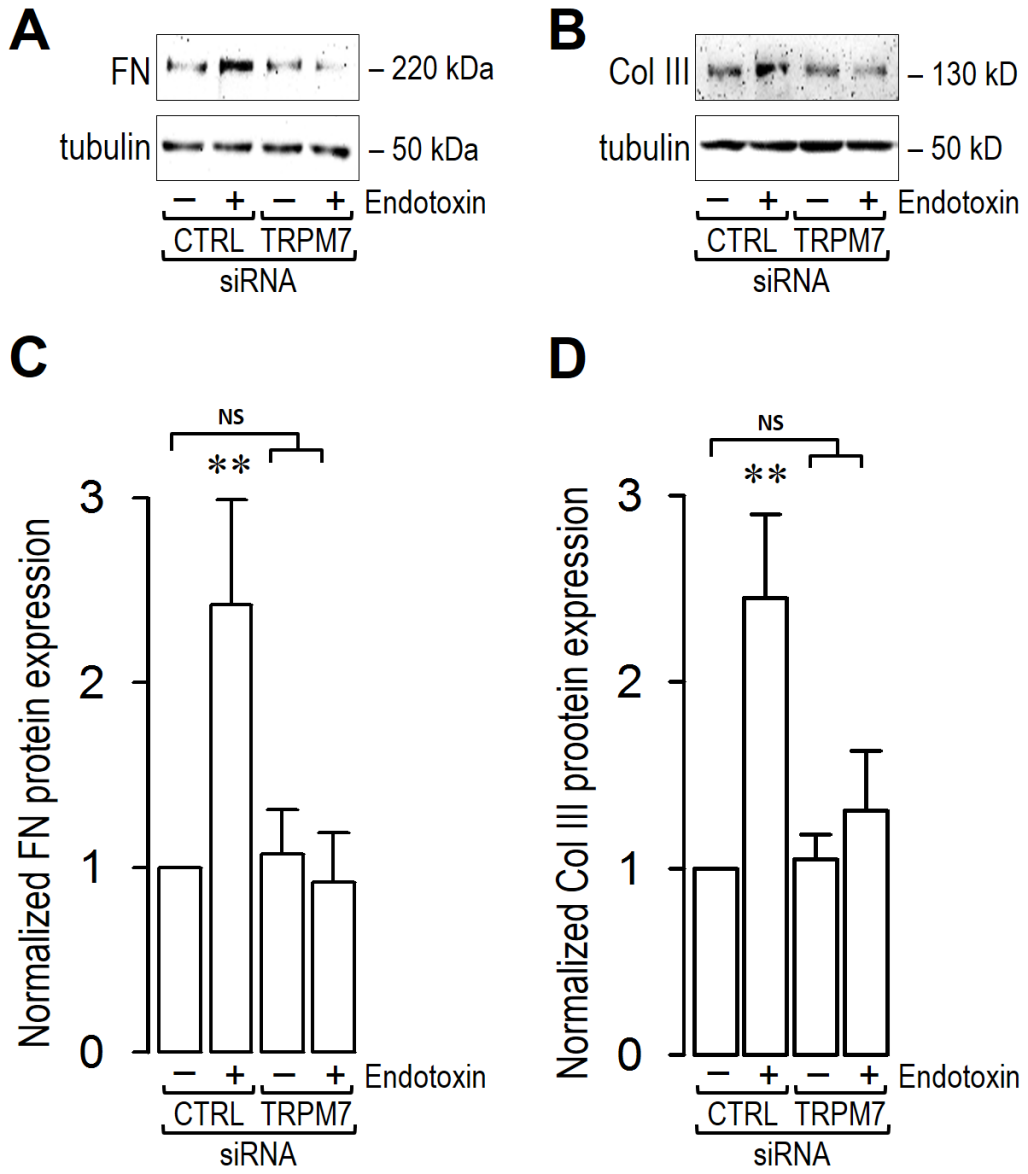
Endotoxin-induced endothelial fibrosis through ECM proteins increase are dependent on TRPM7 expression. ECs were exposed to LPS for 72(A–B) Representative images from western blot experiments performed for detection of ECM proteins fibronectin (FN) (A) and type III collagen (Col III) (B). (C–D) Densitometric analyses of the experiments shown in (A–B) respectively. Protein levels were normalized against tubulin and data are expressed relative to siRNA-CTRL transfected cells without endotoxin condition. Statistical differences were assessed by a one-way analysis of variance (ANOVA) (Kruskal–Wallis) followed by Dunn's post hoc test. *: *p*<0.05 and **: *p*<0.01 against to siRNA-CTRL transfected cells without endotoxin condition. NS: non-significant. Graph bars show the mean ± SD (*N* = 3–6).

Oversecretion of ECM proteins is a key feature of fibrogenesis [41], [42]. Thus, we measured fibronectin and collagen protein levels in the supernatants of EC cultures. LPS-treated ECs transfected with siRNA-CTRL showed an increase in fibronectin (Figure 4A and C) and type III collagen (Figure 4B and D) levels. These results were similar to those previously detected in LPS-treated, non-transfected wild-type endothelial cells [14]. However, the oversecretion of these ECM proteins was abolished by treatment with siRNA-TRPM7. ECs transfected with siRNA-TRPM7 and exposed to LPS did not exhibit any increase in either fibronectin (Figure 4A and C) or type III collagen (Figure 4B and D). These findings are in accord with those obtained using the non-specific TRPM7 blockers Zn^2+^ and Gd^3+^ (Figure S3) [33], [38]–[40]. ECs transfected with siRNA-TRPM7 in the absence of LPS did not show any differences in the expression of ECM markers compared to those observed in vehicle-treated cells transfected with siRNA-CTRL (Figure 4A-D).

**Figure 5:**
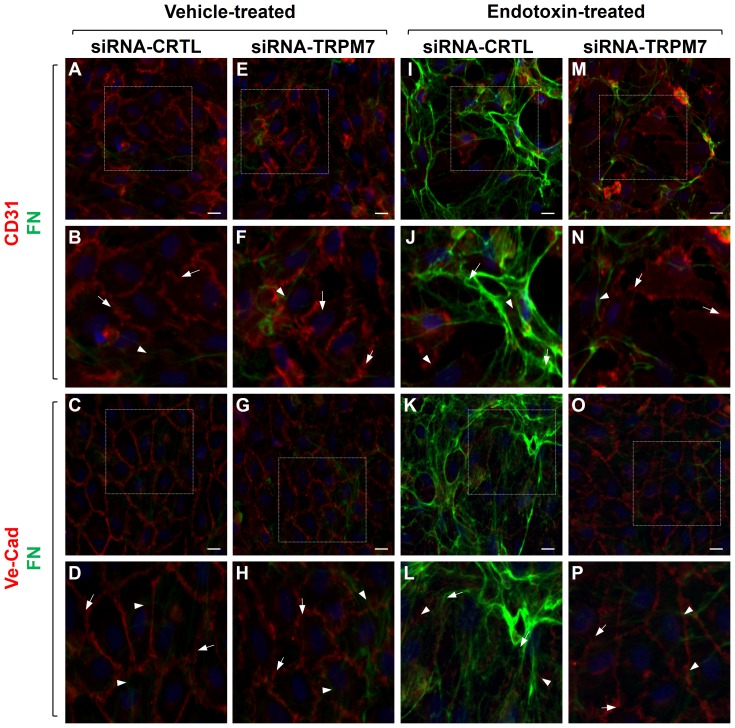
Cellular distribution of ECM proteins involved in endotoxin-induced endothelial fibrosis. Representative images from experiments of vehicle-treated (A–H) or endotoxin (20 μg/mL LPS)-treated (I–P) ECs for 72 h. Endothelial markers CD31 or VE-cadherin (red), and the ECM protein FN (green) were detected. In vehicle-treated cells: the box depicted in (A, C, E, and G) indicates the magnification shown in (B, D, F, and H), respectively. Arrows indicate CD31 (B, F) or VE-cadherin (D, H) labeling at the plasma membrane, whereas arrowheads indicate FN (B, D, F, and H) staining, indicating basal expression of fibrotic markers (B, D). In endotoxin-treated cells: the box depicted in (I, K, M, and O), indicates the magnification shown in (J, L, N, and P) respectively. Arrows indicate FN (J, L, N, and P), whereas arrowheads indicate CD31 (J, N) or VE-cadherin (L, P) staining from residual endothelial marker expression indicating EndMT. Nuclei were stained using DAPI. Bar scale represents 10 μm.

Next, we evaluated the role of TRPM7 expression in the cellular localization and distribution of ECM proteins. ECs transfected with siRNA-CTRL or siRNA-TRPM7 in the absence of endotoxin showed typical CD31 (Figure 5A and E) and VE-cadherin (Figure 5C and G) labeling, localized predominantly at the plasma membrane, whereas FN (Figure 5A, E, C and G) was expressed at low levels. ECs transfected with siRNA-CTRL and exposed to LPS showed increased FN labeling (Figure 5I and K) and decreased expression of CD31 (Figure 5I) and VE-cadherin (Figure 5K), similar to what has previously been reported in endotoxin-treated non-transfected wild-type endothelial cells [14]. In contrast, LPS-treated ECs transfected with siRNA-TRPM7 were resistant to this endotoxin-induced cellular conversion, showing CD31 (Figure 5M) and VE-cadherin (Figure 5O) labeling that was restricted to the plasma membrane, while FN (Figure 5M and O) was weakly expressed.

### TRPM7 mediates the endotoxin-induced increase in intracellular Ca^2+^ in endothelial cells

**Figure 6:**
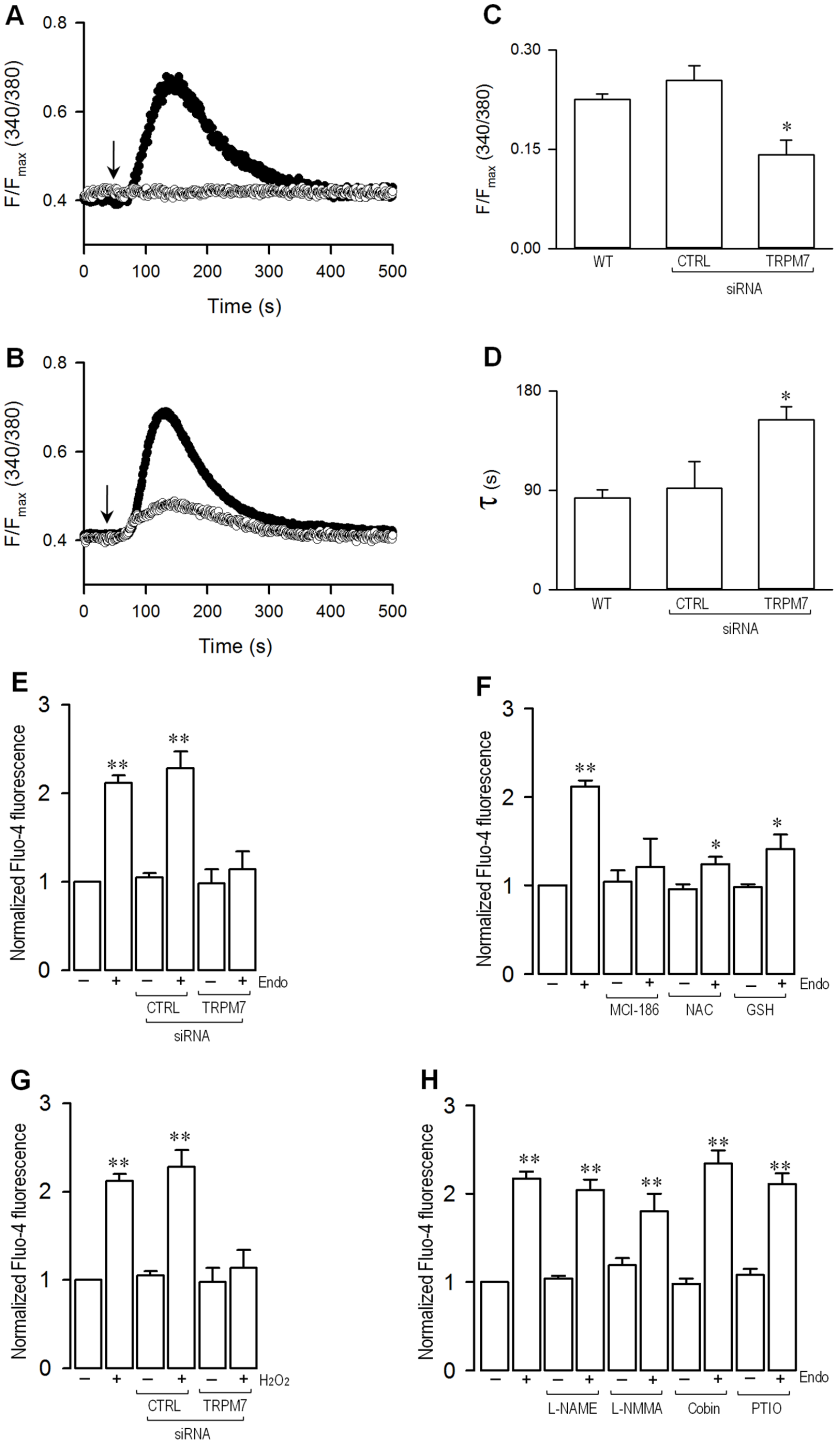
Endotoxin-induced intracellular Ca^2+^ increase is mediated by TRPM7 in endothelial cells. (A) Representative time courses for normalized florescence in ECs loaded with Fura-2 and treated with vehicle (empty circles) or 20 μg/ml LPS (filled circles). The arrow represent the time for external solution change containing vehicle (empty circles) or LPS (filled circles). (B) Representative time courses for normalized florescence in ECs transfected with non-targeting siRNA (siRNA-CTRL, filled circles) or with siRNA for TRPM7 (siRNA-TRPM7, empty circles) and loaded with Fura-2. The arrow indicates the time for LPS application. (C) Summary bar graph for maximal amplitude of normalized florescence in wild type ECs (WT) or transfected with siRNAs. (D) Summary of the data for the time constant (τ) of calcium return to basal levels, obtained after fitting the data to a single exponential for wild type ECs (WT) or transfected with siRNAs. (E–H) Endothelial cells were incubated in the absence (−) or presence (+) of 20 μg/mL endotoxin and the calcium overloading was evaluated by means of the Ca^2+^-sensitive fluorescent dye fluo-4. (E) ECs were transfected with siRNA-TRPM7 and siRNA-CTRL and incubated in the absence (−) or presence (+) of endotoxin. (F) ECs were preincubated with 0.5 mM MCI-186, 1 mM NAC or 2 mM GSH for 1 hr and then incubated in the absence (−) or presence (+) of endotoxin. (G) ECs were transfected with siRNA-TRPM7 and siRNA-CTRL and incubated in the absence (−) or presence (+) of 10 μM H_2_O_2_. (H) ECs were preincubated with 5 mM L-NAME, 1 mM L-NMMA, 50 μM cobinamide, or 500 μM PTIO for 1 hr and then incubated in the absence (−) or presence (+) of endotoxin. Statistical differences were assessed by a one-way analysis of variance (ANOVA) (Kruskal–Wallis) followed by Dunn's post hoc test. *: *p*<0.05; **: *p*<0.01 against vehicle-treated condition. Graph bars show the mean ± SD (*N* = 3–4).

As an increase in the intracellular Ca^2+^ concentration ([Ca^2+^]_i_) has been shown to be necessary for the initiation and development of the fibrotic process [17]–[19], we prompted to investigate whether LPS is able to induce an increase in [Ca^2+^]_i_. To this end, ratiometric Ca^2+^-imaging analyses were performed. Our results showed that ECs exposed to LPS exhibited a transient rise in Ca^2+^ levels (Figure 6A), reaching a maximal level within 90 seconds and slowly returning to basal levels with a half-time constant of 82±7 seconds (Figure 6C). No changes in [Ca^2+^]_i_ were observed in cells treated with vehicle alone (Figure 6A).

To determine whether the TRPM7 channel is involved in the endotoxin-induced intracellular Ca^2+^ increase, we examined whether downregulation of TRPM7 expression via specific siRNA treatment would modify the observed pattern of the increase in intracellular calcium levels. In LPS-treated ECs transfected with siRNA-TRPM7 (Figure 6B), a transient [Ca^2+^]_i_ increment was observed, reaching maximal levels within 90 seconds, and the main differences observed compared to either LPS-treated ECs transfected with siRNA-CTRL (Figure 6B) or LPS-treated non-transfected wild-type ECs (Figure 6A) concerned the [Ca^2+^]_i_ reached (Figure 6C) and the half-time constant for returning to basal levels (Figure 6D). Again, no changes in [Ca^2+^]_i_ were observed in cells treated with vehicle alone (not shown).

Next, we were prompted to study the participation of reactive molecules in the TRPM7-mediated LPS-induced Ca^2+^ level increase. To that end, we monitored changes in the intracellular calcium level by means of the Ca^2+^-sensitive dye Fluo-4 fluorescence. First, we probe that Fluo-4 dye was able to detect changes in the Ca^2+^ level only when endotoxin was added (Figure 6E). Furthermore, the Fluo-4-based assay was able to demonstrate that endotoxin-induced Ca^2+^-increase was dependent on TRPM7 ion channel expression since transfection of siRNA-TRPM7 abolished the calcium increase (Figure 6E). Then, ECs were incubated in the presence of MCI-186, a free radical scavenger which acts over hydroxyl radical, super oxide and peroxynitrite among others. Our results demonstrated that MCI-186 abolished the calcium level increase induced by LPS (Figure 6F). Furthermore, ECs were exposed to the antioxidant N-acetyl cysteine (NAC) or with the reducing agent glutathione in its reduced form (GSH). Results showed that NAC and GSH treatment significantly reduced the LPS-induced calcium increase (Figure 6F). To address definitely the participation of ROS, we exposed ECs to the oxidant agent hydrogen peroxide (H_2_O_2_). Our results showed that ECs exposed to H_2_O_2_ increase the intracellular calcium signal similarly to observed using the endotoxin (Figure 6G). Of note, the H_2_O_2_-induced Ca^2+^-increase was also dependent on TRPM7 ion channel expression because the transfection of siRNA-TRPM7 significantly decreased the H_2_O_2_-induced calcium increase (Figure 6G). Besides, ECs were incubated with the generic inhibitors of all three nitric oxide synthase (NOS) isoforms, L-NG-Nitroarginine Methyl Ester (L-NAME) or with N-monomethyl-L-arginine monoacetate (L-NMMA). Our results demonstrated that the treatment with L-NAME or L-NMMA did not induce any change in the endotoxin-induced Ca^2+^-increase (Figure 6H). In addition, cells were incubated with the nitric oxide (NO) scavengers cobinamide (Cobin) or with 2-Phenyl-4,4,5,5-tetramethylimidazoline-1-oxyl 3-oxide (PTIO), which not affect NO synthesis. Data showed that neither cobinamide nor PTIO modify the endotoxin-induced Ca^2+^-increase (Figure 6H). These results suggest that ROS, but not NO generation, is involved in the TRPM7-mediated intracellular calcium changes induced by endotoxin.

## Discussion

Endothelial dysfunction is a hallmark of the progression of sepsis syndrome and several inflammatory diseases. Because the current available therapies are often not satisfactory, the identification of key proteins involved in these pathologies is essential for improving their treatment. We previously reported that the endotoxin LPS induces endothelial fibrosis [14]. Here, we delved deeper into the molecular mechanism underlying in the endotoxin-induced endothelial fibrosis.

In this study, we demonstrated that the Ca^2+^-permeable channel TRPM7 is a crucial protein in the development of endotoxin-induced endothelial fibrosis. Our results showed that suppression of TRPM7 expression efficiently inhibits the endotoxin-induced conversion of ECs into activated fibroblasts. TRPM7 downregulation prevented the endotoxin-induced decrease of endothelial markers and the increase of fibrotic genes induced by LPS in ECs. Furthermore, TRPM7 downregulation inhibited the endotoxin-induced increase of ECM proteins. Finally, we demonstrated that endotoxin is able to induce a significant increase in the intracellular Ca^2+^ concentration in ECs. This endotoxin-induced Ca^2+^ increase was prevented by inhibition of TRPM7 expression, suggesting that the endotoxin-induced Ca^2+^ increase is mediated by the TRPM7 calcium channel.

In endotoxemia-derived sepsis syndrome, large amounts of LPS are found in the bloodstream, directly interacting with ECs. Because ECs express the toll-like receptor 4 (TLR4) LPS receptor, LPS is able to exert its action in the endothelium of blood vessels. Hence, endotoxin-induced endothelial fibrosis emerges as an important potential mechanism for generating detrimental effects in the organs of patients experiencing endotoxemia-derived sepsis syndrome.

TRPM7 has been found to be involved in a number of human diseases and pathological conditions, including the activation of immune system cells [43], [44], brain ischemia [45], Guamanian lateral sclerosis and Parkinson's [46], atrial fibrillation [29], and cancer [47], [48]. The majority of these pathologies are characterized by an increase in the intracellular level of ROS. Similarly, increases in oxidative stress are a common consequence of inflammatory processes, and it has been reported that LPS induces an increase in intracellular ROS levels in ECs [10], . In addition, TRPM7 activity has been found to be regulated by oxidative stress [33], [38], and the expression of TRPM7 is increased in cells exposed to oxidant agents [39], [50]. Interestingly, endotoxin-induced endothelial fibrosis is prevented by treatment with the reducing agent *N*-acetylcysteine [14]. Considering these findings, it can be suggested that the participation of TRPM7 in the endotoxin-induced endothelial fibrosis could be mediated by the ROS generated by LPS challenge. Further experiments are needed to shed light on this issue.

An interesting feature of TRPM7 is that it contains a C-terminal Ser/Thr kinase domain [36], [51]. As we demonstrated that suppression of TRPM7 expression is necessary for endotoxin-induced endothelial fibrosis in the present study, it is possible that the kinase activity of the channel could also be involved in the induction of endothelial fibrosis. However, further experiments will certainly be needed to verify this hypothesis.

Changes in intracellular Ca^2+^ concentrations are essential for diverse cellular processes to occur normally. However, several lines of evidence suggest that alterations of Ca^2+^ levels are also essential for the development of pathological conditions [20], [52]–[55]. Considering that an increase in intracellular Ca^2+^ is fundamental for fibrogenesis to take place, we sought to study this issue. The endotoxin-induced intracellular Ca^2+^ increase was characterized by a transient elevation of Ca^2+^ and a rapid return to basal calcium levels, suggesting the existence of a negative feedback mechanism regulating the Ca^2+^ increase. The experiments involving siRNA-TRPM7 demonstrated that the endotoxin-induced Ca^2+^ increase was mediated through TRPM7. Experiments performed using MCI-186 and L-NAME suggest that hydroxyl radical, super oxide and peroxynitrite, but not nitric oxide, could be involved in the endotoxin-induced Ca^2+^ increase. Furthermore, since the inhibition of the endotoxin-induced Ca^2+^ increase produced by NAC and GSH, it is possible to hypothesize that the oxidative modifications produced in TRPM7 could be performed in thiol groups from cysteine residues. These findings suggest either that TRPM7 directly participates in the mechanism regulating the endotoxin-induced Ca^2+^ increase or that other TRPM7-related proteins are involved in the regulation of the TRPM7-mediated calcium influx. In this context, a non-selective cation channel, TRPM4, has been shown to be involved in the regulation of intracellular calcium overloading and oscillations by controlling the plasma membrane potential [56]–[58]. Thus, TRPM4 activity indirectly controls the calcium influx, which is mediated by additional calcium channels regulating intracellular calcium homeostasis to promote physiological and pathological processes.

ECM proteins are produced and secreted in balance with their degradation in healthy cells, whereas during fibrosis, activated fibroblasts oversecrete ECM proteins, thereby overwhelming the capability for ECM degradation [59], [60]. Thus, increases in ECM proteins are a sign that a fibrogenic process has taken place. On the other hand, ECM proteins may play a role in bacterial adherence and invasion to promote endotoxin-mediated endothelium damage [61]–[63], as overexpression of ECM proteins facilitates the pathogenic mechanism. The fact that inhibition of the TRPM7 channel abolished the endotoxin-induced increase in ECM expression in ECs indicates that this channel is a key protein involved in the progression of endothelial fibrosis under endotoxemia-like conditions. Further studies must be performed to evaluate whether TRPM7 may be useful as a therapeutic tool.

Taken together, these findings demonstrated that the TRPM7 channel plays a critical role in the mechanism underlying endotoxin-induced endothelial fibrosis. Thus, TRPM7 emerges as a novel target for drug design to improve current treatments against endotoxemia-derived sepsis syndrome and further inflammatory diseases.

## Supporting Information

Figure S1 **Primary HUVEC cultures were subjected to immunocytochemistry experiments to identify ECs as VE-Cad positive cells (VE-Cad^+^) and non-endothelial as VE-Cad negative cells (VE-Cad^−^).** Data are expressed as percentage of total cells counted. Several independent experiments were counted (N  =  10). Statistical differences were assessed by student's t-test (Mann-Whitney). ***: *p*<0.0001.
(PDF)

Figure S2 **Endotoxin-induced endothelial fibrosis through changes in endothelial and fibrotic markers are inhibited by using the non-specific TRPM7 blocker Zn^2+^ and Gd^3+^.** (**A**–**D**) ECs were exposed to LPS for 72 h in the presence of Zn^2+^ (**A**–**B**) or Gd^3+^ (**C**–**D**), and protein expression of endothelial marker CD31 (**A** and **C**) and fibrotic markers α-SMA (**B** and **D**). Statistical differences were assessed by a one-way analysis of variance (ANOVA) (Kruskal–Wallis) followed by Dunn's post hoc test. **: *p*<0.01 against to siRNA-CTRL transfected cells without endotoxin condition. NS: non-significant. Graph bars show the mean ± SD (*N*  =  3).
(PDF)

Figure S3 **Endotoxin-induced endothelial fibrosis through ECM proteins increase are inhibited by using the non-specific TRPM7 blocker Zn^2+^ and Gd^3+^.** (**A**–**D**) ECs were exposed to LPS for 72 h in the presence of Zn^2+^ (**A**–**B**) or Gd^3+^ (**C**–**D**), and ECM proteins fibronectin (FN) (**A** and **C**) and type III collagen (Col III) (**B** and **D**). Statistical differences were assessed by a one-way analysis of variance (ANOVA) (Kruskal–Wallis) followed by Dunn's post hoc test. **: *p*<0.01 against to siRNA-CTRL transfected cells without endotoxin condition. NS: non-significant. Graph bars show the mean ± SD (*N* = 3).
(PDF)

Table S1 **Primary and secondary antibodies used in western blot experiments.**
(PDF)

Table S2 **Primary and secondary antibodies used in immunocytochemistry experiments.**
(PDF)

Methods S1 **Supporting expanded methods.**
(PDF)
