# Supplementary material for: Endotoxin Induces Fibrosis in Vascular Endothelial Cells through a Mechanism Dependent on Transient Receptor Protein Melastatin 7 Activity
Source: PLoS One. 2014 Apr 7;9(4):e94146. doi: 10.1371/journal.pone.0094146 (PMC3978016; doi:10.1371/journal.pone.0094146)
Supplement: Figure S1 — Primary HUVEC cultures were subjected to immunocytochemistry experiments to identify ECs as VE-Cad positive cells (VE-Cad+) and non-endothelial as VE-Cad negative cells (VE-Cad−). Data are expressed as percentage of total cells counted. Several independent experiments were counted (N = 10). Statistical differences were assessed by student's t-test (Mann-Whitney). ***: p<0.0001. (PDF) [file pone.0094146.s001.pdf]

Figure S1

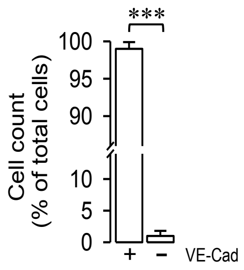

Figure S1. Primary HUVEC cultures were subjected to immunocytochemistry experiments to identify ECs as VE-Cad positive cells (VE-Cad^{+}) and non-endothelial as VE-Cad negative cells (VE-Cad^{-}). Data are expressed as percentage of total cells counted. Several independent experiments were counted (N = 10). Statistical differences were assessed by student's t-test (Mann-Whitney). ***: $p < 0.0001$.
