# Supplementary material for: Endotoxin Induces Fibrosis in Vascular Endothelial Cells through a Mechanism Dependent on Transient Receptor Protein Melastatin 7 Activity
Source: PLoS One. 2014 Apr 7;9(4):e94146. doi: 10.1371/journal.pone.0094146 (PMC3978016; doi:10.1371/journal.pone.0094146)
Supplement: Figure S2 — Endotoxin-induced endothelial fibrosis through changes in endothelial and fibrotic markers are inhibited by using the non-specific TRPM7 blocker Zn2+ and Gd3+. (A–D) ECs were exposed to LPS for 72 h in the presence of Zn2+ (A–B) or Gd3+ (C–D), and protein expression of endothelial marker CD31 (A and C) and fibrotic markers α-SMA (B and D). Statistical differences were assessed by a one-way analysis of variance (ANOVA) (Kruskal–Wallis) followed by Dunn's post hoc test. **: p<0.01 against to siRNA-CTRL transfected cells without endotoxin condition. NS: non-significant. Graph bars show the mean ± SD (N = 3). (PDF) [file pone.0094146.s002.pdf]

Figure S2

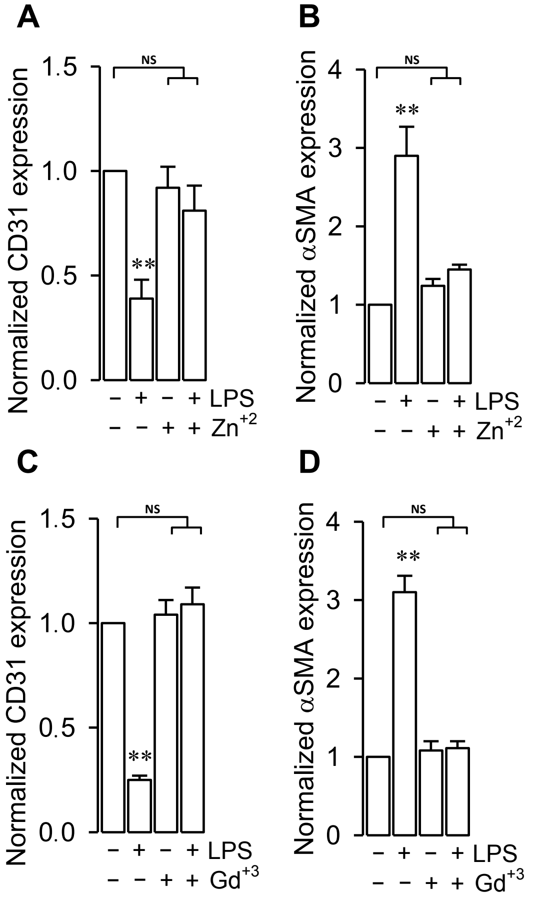

Figure S2. Endotoxin-induced endothelial fibrosis through changes in endothelial and fibrotic markers are inhibited by using the non-specific TRPM7 blocker Zn^{2+} and Gd^{3+}. (**A**–**D**) ECs were exposed to LPS for 72 h in the presence of Zn^{2+} (**A**–**B**) or Gd^{3+} (**C**–**D**), and protein expression of endothelial marker CD31 (**A** and **C**) and fibrotic markers α-SMA (**B** and **D**). Statistical differences were assessed by a one-way analysis of variance (ANOVA) (Kruskal–Wallis) followed by Dunn's post hoc test. **: $p < 0.01$ against to siRNA-CTRL transfected cells without endotoxin condition. NS: non-significant. Graph bars show the mean ± SD ($N = 3$).
