# Supplementary material for: Endotoxin Induces Fibrosis in Vascular Endothelial Cells through a Mechanism Dependent on Transient Receptor Protein Melastatin 7 Activity
Source: PLoS One. 2014 Apr 7;9(4):e94146. doi: 10.1371/journal.pone.0094146 (PMC3978016; doi:10.1371/journal.pone.0094146)
Supplement: Table S1 — Primary and secondary antibodies used in western blot experiments. (PDF) [file pone.0094146.s004.pdf]

Table S1. Primary and secondary antibodies used in western blot experiments.

Primary Ab	*Dilution*	*Source*	*Incubation time*	*Incubation temperature*	*Brand*
**CD31**	1:1000	Mouse	2 h	RT	Dako
**VE-cadherin**	1:500	Goat	2 h	RT	Santa Cruz
**α-SMA**	1:1000	Rabbit	2 h	RT	Millipore
**FSP-1**	1:500	Mouse	2 h	RT	Abcam
**Type III collagen**	1:3000	Rabbit	2 h	RT	Rockland
**Fibronectin**	1:6000	Rabbit	2 h	RT	Sigma-Aldrich
**α-tubulina**	1:5000	Mouse	2 h	RT	Sigma-Aldrich

Secondary Ab	*Dilution*	*Source*	*Incubation time*	*Incubation temperature*	*Brand*
**Anti- Mouse HRP**	1:5000	Mouse	2 h	RT	Millipore
**Anti- Rabbit HRP**	1:5000	Goat	2 h	RT	Millipore
**Anti- Goat HRP**	1:6000	Rabbit	2 h	RT	Abcam
