# Supplementary material for: Endotoxin Induces Fibrosis in Vascular Endothelial Cells through a Mechanism Dependent on Transient Receptor Protein Melastatin 7 Activity
Source: PLoS One. 2014 Apr 7;9(4):e94146. doi: 10.1371/journal.pone.0094146 (PMC3978016; doi:10.1371/journal.pone.0094146)
Supplement: Table S2 — Primary and secondary antibodies used in immunocytochemistry experiments. (PDF) [file pone.0094146.s005.pdf]

Table S2. Primary and secondary antibodies used in immunocytochemistry experiments.

Primary Ab	*Dilution*	**Source**	*Incubation time*	*Incubation temperature*	*Brand*
**CD31**	1:200	Mouse	ON	4^oC	Dako
**VE-cadherin**	1:100	Goat	ON	4^oC	Santa Cruz
**α-SMA**	1:500	Rabbit	ON	4^oC	Millipore
**FSP-1**	1:100	Mouse	ON	4^oC	Abcam
**Fibronectin**	1:400	Rabbit	ON	4^oC	Sigma-Aldrich

Secondary Ab	*Dilution*	*Source*	*Incubation time*	*Incubation temperature*	*Brand*
**Anti- rabbit Alexa-488**	1:250	Goat	2 h	RT	Invitrogen
**Anti- goat Alexa-594**	1:250	Donkey	2 h	RT	Invitrogen
**Anti- mouse Alexa-488**	1:250	Goat	2 h	RT	Invitrogen
**Anti- mouse Alexa-594**	1:250	Goat	2 h	RT	Invitrogen
