# Supplementary material for: Endotoxin Induces Fibrosis in Vascular Endothelial Cells through a Mechanism Dependent on Transient Receptor Protein Melastatin 7 Activity
Source: PLoS One. 2014 Apr 7;9(4):e94146. doi: 10.1371/journal.pone.0094146 (PMC3978016; doi:10.1371/journal.pone.0094146)
Supplement: Methods S1 — Supporting expanded methods. (PDF) [file pone.0094146.s006.pdf]

Supporting Information

Endotoxin induces fibrosis in vascular endothelial cells through a mechanism dependent on transient receptor protein melastatin 7 activity.

Cesar Echeverría, Ignacio Montorfano, Tamara Hermosilla, Ricardo Armisén, Luis A. Velásquez, Claudio Cabello-Verrugio, Diego Varela, and Felipe Simon

Supporting expanded Methods

Ethics Statement

The investigation conforms with the principles outlined in the Declaration of Helsinki. The Commission of Bioethics and Biosafety of Universidad Andres Bello also approved all experimental protocols. Human umbilical cord were obtained from patients after written patient's informed consent. The individual in this manuscript has given written informed consent (as outlined in PLOS consent form) to publish these case details.

Primary cell culture

Human umbilical vein endothelial cells (HUVEC) were isolated by collagenase (0.25 mg/mL) digestion from freshly obtained umbilical cord veins from normal pregnancies, after patient's informed consent. Cells were grown in gelatin-coated dishes at 37°C in a 5%:95% CO_2:air atmosphere in medium 199 (Sigma, MO), containing 100 μg/mL endothelial cell growth supplement (ECGS) (Sigma), 100 μg/mL heparin, 5 mmol/L D-glucose, 3.2 mmol/L L-gutamine, 10% fetal bovine serum (FBS) (GIBCO, NY), and 50 U/mL penicillin-streptomycin (Sigma).

Small interfering RNA against TRPM7 and transfection

SiGENOME SMARTpool siRNA (four separated siRNAs per human TRPM7 transcript) were purchased from Dharmacon (Dharmacon, Lafayette, CO). The following siRNA were used: human TRPM7 (siRNA-TRPM7) and non-targeting siRNA (siRNA-CTRL) used as a control. In brief, HUVEC were plated overnight in 24-well plate and then transfected with 5 nmol/L siRNA using DharmaFECT 4 transfection reagent (Dharmacon) used according to the manufacturer's protocols in serum-free medium for 6 hours. After 24 to 48 transfection, experiments were performed.

Western blot procedures

Vehicle-treated or LPS-treated ECs were lysed in cold lysis buffer [150 mmol/L NaCl, 1 mmol/L EGTA, 50 mmol/L Tris, pH 7.4, 1% glycerol, 1% Triton X-100, 10 mmol/L NaF, 20 mmol/L NaPi, and protease inhibitor cocktail (Sigma)] and centrifuged (10,000×g for 15 min at 4°C), and then proteins were extracted. Supernatants were

collected and stored in the same lysis buffer. Protein extract and supernatant were subjected to 12% (for FSP-1), 10% (for CD31, VE-cadherin, α-SMA, and tubulin), or 8% (for Col III and FN) SDS-PAGE. Resolved proteins were transferred to a nitrocellulose or PVDF membrane and non-specific binding was blocked using 5% BSA in PBS for 1 h at pH 7.4. The blocked membrane was incubated with the appropriate primary antibody, washed twice, and incubated with a secondary antibody. Bands were revealed using a peroxidase-conjugated IgG antibody. Tubulin was used as a loading control. Peroxidase activity was detected through enhanced chemiluminescence (Bio-Rad, CA) and images were acquired using Fotodyne FOTO/Analyst Luminary Workstations Systems (Fotodyne, Inc., Hartland, WI). Protein content was determined by densitometric scanning of immunoreactive bands and intensity values were obtained by densitometry of individual bands normalized against tubulin. For a detailed list of antibodies used, see Supplementary Table 1.

Fluorescent immunocytochemistry

ECs were washed twice with PBS and fixed with 3.7% PFA for 30 min at RT, treated with 50 mmol/L NH_4Cl for 15 min at RT, permeabilized with 0.1% Triton X-100 in PBS for 30 min at RT, and blocked for 2 h at RT with 3% BSA in PBS. The cells were subsequently washed again and incubated with the first primary antibodies. Then, cells were washed twice and incubated with the first secondary antibodies. For immunofluorescent double staining, the cells were washed with PBS twice and the above staining procedure was repeated for the second set of primary and second secondary antibodies. Samples were mounted with ProLong Gold antifade mounting medium with DAPI (Invitrogen). For a detailed list of antibodies used see Supplementary Table 2.

Calcium Imaging

Plated ECs were mounted in a perfusion chamber on the stage of an inverted microscope (Olympus IX-81, UPLFLN 40XO 40 x/1.3 oil-immersion objective). Cells were incubated with 1 μM Fura-2 AM (Molecular Probes) for 30 min and then washed with Hank's solution (mM): 116 NaCl, 5.4 KCl, 2 $CaCl_2$, 0.8 $MgSO_4$, 0.8 NaH_2P, 5 glucose and 20 Hepes, pH 7.4, adjusted with Tris. Fura-2 was alternately excited at 340 and 400 nm, and the fluorescence filtered at 510 nm was collected and recorded at 5 Hz using a CCD-based imaging system (Olympus DSU) running CellR software (Olympus). At the end of each experiment, maximal fluorescence was obtained by treating the cell with 1 μM ionomicin. For every experiment, signals were recorded and the background intensity was subtracted, using a same-size region of interest outside the cells [1]. Results are expressed as the ratio between the 340 nm and 400 nm (R340/400) signals.

Measurement of [Ca^{2+}] by flow cytometry

ECs were harvested with trypsin/EDTA, washed twice in ice-cold PBS, resuspended and loaded with the Ca^{2+}-sensitive cell permeant dye Fluo-4 (5 μM) for 15-30 min at room temperature in the dark. Then, cells were exposed to LPS for 90 s and analyzed immediately by flow cytometry (FACSCanto, BD Biosciences, San José, CA). ECs were transfected (with siRNA-TRPM7 and siRNA-CTRL) or preincubated with L-NAME, MCI-186, NAC, and GSH. Experiments were performed and then intracellular calcium levels were measured using Fluo-4 dye. Using the FACSDiva software, a population of red-positive cells (transfected cells) was defined, and calcium levels for this population were

analyzed. A minimum of 10,000 cells/sample were analyzed. Cellular dye intensity analysis was performed using FACSDiva software v4.1.1 (BD Biosciences).

Reagents

Lipopolysaccharide from *E. coli* was purchased from Sigma (0127:B8). Fura-2 and Fluo-4 were purchased from Invitrogen. L-NAME, cobinamide, NAC and GSH were purchased from Sigma. PTIO and L-NMMA were purchased from Tocris Bioscience (Bristol, UK). MCI-186 was purchased from Calbiochem (San Diego, CA). Human TGF-β1 and TGF-β2 were purchased from R&D Systems. Buffers and salts were purchased from Merck Biosciences (Darmstadt).

Data analysis

All results are presented as the mean ± SD. ANOVA followed by the Bonferroni or Dunn's *post hoc* tests were used and considered significant at $p<0.05$.

References to Supplementary Methods

1. Hermosilla T, Moreno C, Itfinca M, Altier C, Armisen R, et al. (2011) L-type calcium channel beta subunit modulates angiotensin II responses in cardiomyocytes. Channels (Austin) 5: 280-286.
